# Clinical factors contributing to high cost hospitalizations in a Canadian tertiary care centre

**DOI:** 10.1186/s12913-017-2746-6

**Published:** 2017-11-25

**Authors:** Babak Rashidi, Daniel M. Kobewka, David J. T. Campbell, Alan J. Forster, Paul E. Ronksley

**Affiliations:** 1 Department of Medicine, University of Ottawa, Ottawa, ON Canada; 2 Department of Epidemiology and Community Medicine, University of Ottawa, Ottawa, ON Canada; 3 Department of Medicine, Cumming School of Medicine, University of Calgary, Calgary, AB Canada; 4 Department of Clinical Epidemiology, Ottawa Hospital Research Institute, Ottawa, ON Canada; 5 Department of Community Health Sciences, Cumming School of Medicine, University of Calgary, HSC G239, 3330 Hospital Drive NW, Calgary, AB T2N 4N1 Canada

**Keywords:** Administrative data, Healthcare cost, Hospitalization

## Abstract

**Background:**

Like much of the developed world, healthcare costs in Canada are rising. A small proportion of patients account for a large proportion of healthcare spending and much of this spending occurs in acute care settings. The purpose of our study was to determine potentially modifiable factors related to care processes that contribute to high-cost admissions.

**Methods:**

Using a mixed-methods study design, factors contributing to high-cost admissions were identified from literature and case review. We defined pre- and post-admission factors contributing to high-cost admissions. Pre-admission factors included reason for admission (e.g. complex medical, elective surgery, trauma, etc.). Post-admission factors included medical complications, disposition delays, clinical services delays, and inefficient clinical decision-making. We selected a random sample of admissions in the top decile of inpatient cost from the Ottawa Hospital between January 1 and December 31, 2010. A single reviewer classified cases based on the pre- and post-admission factors. We combined this information with data derived from the Ottawa Hospital Data Warehouse to describe patient-level clinical and demographic characteristics and costs incurred.

**Results:**

We reviewed 200 charts which represents ~5% of all high cost admissions within the Ottawa Hospital in 2010. Post-admission factors contributing to high-cost admissions were: complications (60%), disposition delays (53%), clinical service delays (39%), and inefficient clinical decision-making (13%). Further, these factors varied substantially across service delivery lines. The mean (standard deviation (SD)) cost per admission was $49,923 CDN ($45,773). The most common reason for admission was “complex medical” (49%) and the overall median (IQR) length of stay was 27 (18–48) days. Approximately 1 in 3 high cost admissions (29%) included time in the intensive care unit (ICU).

**Conclusions:**

While high cost admissions often include time in ICU and have long lengths of stay, a substantial proportion of costs were attributable to complications and potentially preventable delays in care processes. These findings suggest opportunities exist to improve outcomes and reduce costs for this diverse patient population.

## Background

Healthcare accounts for 11% of all government spending in Canada [1]. Acute care spending by hospitals accounts for the largest proportion of health spending (~30%) [1]. As costs continue to rise, there is increasing pressure to derive greater value for money. Studies of acute care spending have found that a small number of patients account for a large proportion of healthcare costs with 5% of the general population accounting for more than half of healthcare spending [2–6]. Patients who utilize greater resources are often older, have more comorbidities, and are more likely to experience complications while in hospital, compared to lower cost patients [4, 6–10].

High-cost patients have been characterized previously but the majority of these studies have used administrative data and lack the clinical detail that can be obtained from medical chart review. Recent work has shown that many high-cost patients accrue the bulk of their costs during a single hospital admission [7]. Therefore, detailed characterization and analysis of individual high cost admissions may yield valuable insight into potential cost saving measures. Further, few studies have evaluated how much of the inpatient spending in this high-cost group is due to potentially modifiable aspects of care.

Our objective was to understand how potentially modifiable care processes contribute to total cost among a sample of high cost hospitalizations. Unlike many other studies, which look at the cost linked to specific medical conditions, we studied all high cost patients allowing us to identify potential factors common to this subset of hospitalized patients. In order to do this we performed qualitative documentary analysis of in-patient medical records to explore and characterize factors that inflated costs and then performed a quantitative description of the sample using hospital administrative data.

## Methods

### Overall mixed methods study design

To accomplish our study objective, we used an exploratory sequential mixed methods design, with priority given to the quantitative component. This design allowed for an initial qualitative phase to inform a quantitative phase that generalized the findings of the qualitative work. Beginning this study with a qualitative strand enabled us to verify that the factors previously identified in the published literature were again important contributors in this particular population – and to give us confidence that the coding template we used was complete and representative. This study was approved by the Ottawa Health Sciences Network Research Ethics Board and granted waiver of patient consent.

### Study population and data sources

The study took place at the Ottawa Hospital, a large multi-site tertiary care teaching hospital with 1065 beds. Patients’ medical records were used for qualitative documentary analysis. The hospital’s data warehouse was used for clinical, demographic, and case costing information.

### Qualitative phase

To explore the various contributors to high cost hospitalizations, we analyzed documentary sources (patient medical charts) using content analysis techniques.

#### Sampling

We utilized a random purposeful sampling technique to determine which charts to review [11]. We used The Ottawa Hospital’s Data Warehouse to identify all individuals (regardless of age) with one or more inpatient admissions that started between January 1, 2010 and December 31, 2010 (*n* = 44,812 patients). We followed all patients through to the end of their admission. Costs for each patient were identified within the case costing system of the Ottawa Hospital Data Warehouse. The Ottawa Hospital employs a standardized case costing methodology developed by the Ontario Case Costing Initiative [12] and is based on the Canadian Institute for Health Information (CIHI) Management Information Systems guidelines and the Ontario Healthcare Reporting Standards [13]. The primary purpose of case costing standards is to ensure comparability across Canadian hospitals.

Costs for each admission were calculated as the sum of costs in 11 resource-specific categories (e.g. nursing costs, laboratory costs, pharmacy costs, operating room costs). These were divided into direct costs for patient care (e.g. nursing, laboratory, and pharmacy), and indirect costs not immediately affecting patient care (e.g. heating, cleaning, and other building/administrative costs). Using the distribution of total accumulated cost (direct + indirect) per patient, admissions with costs in the upper 10th percentile were defined as ‘high cost admissions’ (*n* = 4758). A random sample of 200 of these high cost admissions (representing ~5% of all high cost admissions) was selected for our study population. Because admissions were the unit of analysis, a given patient could be included in the sample more than once if they had multiple admissions that were classified as high cost during the observation period.

#### Analysis

To determine which post-admission factors contributed to cost we used a directed approach to content analysis – where content analysis techniques were applied deductively [14, 15]. The first step was to create a preliminary coding template from the literature and existing theories of contributors to high cost hospitalizations. We identified three potential factors that contribute to high cost hospitalizations. These included:

1. Unforeseen in-hospital complications [16–18] defined as medical problems not present on admission, which increased length of stay by greater than 24 h;
2. Delays in patient disposition [19, 20] defined as a delay in finding an appropriate discharge location after acute medical issues had resolved resulting in a prolonged length of stay of greater than 24 h;
3. Service delays [21] defined as a delay in the provision of required hospital services by greater than 24 h, which likely contributed to an increase in overall cost.

These three overarching themes comprised our initial coding template and we planned to add new themes to the coding template as required by the data. Initially, a subset of 50 high cost admissions was analyzed by three reviewers (BR, AF, PR) using our preliminary coding template. The reviewers met to discuss their coding and to reach consensus on the classification and definition of each content category. Once consensus was reached on the initial 50 charts, a single reviewer (BR) analyzed the remaining high cost admissions, classifying them according to the identified themes. The identification of categories was aided by the fact that the primary reviewer (BR) is a hospital-based clinician and is experienced in reading and interpreting medical charts.

In order to determine pre-admission factors that impacted total cost we used a modified version of the admission-type classification. The classification was created using chart review and clinical experience of the reviewers. The classification along with definitions can be found in Table 1. Once our final coding of factors contributing to high cost hospitalizations was completed using content analysis, we then launched the quantitative phase of our study.

**Table 1:** Classification and definition of admission type

Admission type	Definition
Complex medical	Patient admitted for one or more medical issues, not requiring surgery, chemotherapy, or radiation
Emergent surgery	Patient admitted for and received emergency unscheduled surgical intervention, not related to a trauma
Elective surgery	Patient admitted for previously scheduled correction of a medical problem
Trauma	Patient admitted for management of a traumatic injury (including falls from standing height)
Cancer with chemotherapy/radiation	Patient admitted for treatment of a cancer with chemotherapy or radiation
Social	Patient admitted for purely disposition issues
Maternal	Patient admitted for management of a peri-partum/pregnancy related issue

### Quantitative phase

We used a retrospective observational design to identify the prevalence of the various contributing factors in high cost hospitalizations and associations between these factors and clinical/socio-demographic characteristics.

#### Key variables

*Clinical and encounter characteristics*: Patient and encounter characteristics were extracted from the hospital’s data warehouse – a relational database containing information from several of The Ottawa Hospital’s information systems including the patient registration system, clinical data repository, case-costing system, and patient abstracts from multiple encounter types. Specifically, we extracted data on admission route (emergent, urgent, or elective), total length of stay (sub-divided between acute, alternate level of care (ALC) defined as days in hospital because no appropriate discharge location was available, and intensive care unit (ICU) days), discharge disposition, and the number of inpatient admissions and hospital days within a one-year period following discharge from the high cost admission. Patient-level characteristics were also extracted from the data warehouse including patient age at admission, sex, and comorbidity defined using the derived Elixhauser comorbidity score (a score calculated based on the number of unique medical conditions a patient has from a list of 31 conditions identified within inpatient administrative records) [22, 23]. Coded inpatient information within the hospital data warehouse employs the same data quality standards as the CIHI Discharge Abstracts Database and is based on the International Statistical Classification of Diseases and Related Health Problems, 10th revision – Canada (ICD-10-CA).

#### Statistical analyses

Descriptive statistics of patient and encounter-level characteristics were summarized using proportions, medians with inter-quartile range (IQR), and means with standard deviation (SD) where appropriate. The proportion of admissions with each factor contributing to the high cost admission was also calculated. A post-hoc subgroup analysis was also performed to determine if there were differences in high cost hospitalizations across the four most common admission types (complex medical, elective surgery, emergent surgery, trauma). Stratum-specific estimates were compared using t-tests, chi-squared tests, and the Kruskal-Wallis test for skewed variables. All quantitative analyses were conducted using SAS 9.3 statistical software (SAS Institute Inc., Cary, NC).

## Results

### Qualtitative phase

Our content analysis of 200 patient medical charts supported the coding template that we had designed a priori from our review of the literature. However, we identified one salient additional contributor which was not included in our initial template: medical errors or delays in clinical decision-making that prolonged stays and drove costs. This was operationalized as a missed diagnosis, sequential single systems approach, or narrow differential diagnosis considered by healthcare providers that prolonged a hospital length of stay by 24 h or more. Our final coding scheme was comprised of four key features which contribute to an individual’s inpatient costs. These are: (1) in-hospital complications; (2) delays in patient disposition; (3) service delays; and (4) inefficient clinical decision-making. Below we provide pseudonymised narrative case vignettes representative of each of these contributors.

*In-hospital complications* were medical problems not present on admission, which increased length of stay by greater than 24 h. The following encounter demonstrates a case where medical complications contributed to hospital costs:

A 69 year-old woman was admitted for induction chemotherapy for newly diagnosed acute myeloid leukemia. After about a week in hospital, she developed febrile neutropenia due to a central line infection. She was treated with broad-spectrum antibiotics, with empiric antifungal agents added later due to ongoing fevers. Two weeks into the admission, she developed acute kidney injury necessitating placement of a hemodialysis catheter. Following the line insertion the patient developed an unstable tachyarrhythmia resulting in hypotension and shock. She was transferred to ICU where she required vasopressors and intubation. She was electrically cardioverted with no improvement in blood pressure. Soon after admission to ICU, a family meeting was held and it was decided to change goals of care to focus on comfort measures. The patient died later that day after withdrawing life-sustaining therapies.

*Delays in patient disposition* were identified when length of stay was prolonged by greater than 24 h due to a delay in finding an appropriate discharge location after acute medical issues had resolved. Anecdotally, most patients who required additional ongoing care on discharge from hospital (i.e. homecare, long-term care) had a delay in discharge due to issues with disposition. For example:

An 86 year-old woman was transferred from a community hospital for fluid overload secondary to end stage renal disease. She was started on hemodialysis and her volume status improved in less than one week. The remainder of her one-month stay was spent waiting for transfer to a long-term care facility that could provide her with regular dialysis. Her application to long-term care had been started in the community hospital more than a month prior when it was first identified she would likely require more care.

*Service delays* were defined as a delay in the provision of required hospital services by greater than 24 h, which likely contributed to an increase in overall cost. Anecdotally, the leading two causes of service delays were due to delays in transferring patients between various acuities of care (i.e. ICU to regular ward, regular ward to rehabilitation), and in delays due to waiting for allied health (i.e. physiotherapy, speech-language pathology). An example where service delays increased the cost of a hospitalization:

A 77 year-old man was admitted for unilateral lower extremity weakness resulting in falls. Neuro-imaging with CT and MRI revealed a tumor in the left temporal lobe of the brain. The admitting team decided to obtain a biopsy, however there was a delay of four days before the patient was taken to the operating room for the biopsy. Following the biopsy, there was a delay of five days while waiting for biopsy results. Radiation oncology was subsequently consulted, and a decision was made to pursue treatment with palliative radiation therapy. The patient waited for one week before he was transferred to a different campus of the hospital for initiation of treatment.

*Inefficient clinical decision-making* was when the length of stay was prolonged by greater than 24 h because of a missed diagnosis, sequential single systems approach, or narrow differential diagnosis considered by healthcare providers. An example of a case where inefficient decision-making led to increased cost:

A 60 year-old man was transferred from a community hospital for resection of an oral cancer. He had been initially admitted to a community hospital for a hip fracture requiring surgery. There he had been treated with warfarin as prophylaxis for venous thromboembolic disease; heparins were not used due to a local reaction to injections. At our hospital, his surgery was delayed by one week because of excessively thinned blood (elevated international normalized ratio (INR)). The hematology inpatient service was consulted and recommended administering parenteral vitamin K to reverse his coagulopathy – which took several days.

For illustrative purposes, we chose the above vignettes for each of our contributors to length of stay as they are clear examples of a single contributing factor. However, the majority of charts that we reviewed contained multiple contributors as exemplified by the following case:

An 83 year-old woman was admitted for fatigue, peripheral edema, and declining function at her retirement home. Although one member of the attending service noted that the most likely cause of the edema was poor nutrition, the team undertook an extensive series of investigations for conditions with much lower clinical likelihood, beginning with an echocardiogram to investigate for heart failure. When the echocardiogram was reported as normal, urinary biochemistry studies to pursue unlikely diagnoses such as carcinoid tumour was undertaken. After one week of serial testing, the inpatient gastroenterology team was consulted for assistance [inefficient clinical decision-making]. The GI team elected to perform an esophago-gastro-duodenoscopy, which showed delayed gastric emptying; there was a one-week wait for this test due to a lack of urgent indication, with the endoscopy suite running at full capacity [service delay]. After being diagnosed with delayed gastric emptying and malnutrition, the patient was seen by the geriatric rehabilitation service who determined that she was not a suitable candidate. The decision was made to transfer her to long-term care, with a subsequent one-month wait in the acute care hospital [disposition delay].

Very few high-cost cases had none of the four contributors identified above. An example of such a case:

A 66 year-old female was admitted for elective endovascular repair of an infra-renal abdominal aortic aneurism measuring 5.9 cm. The patient spent a few days in hospital for recovery, and was subsequently discharged with no complications.

#### Quantitative phase

Using costing details from all admissions between January 1st 2010 and December 31st 2010, the upper decile of admissions incurred a cost $28,000 or more. A random sample of 200 of these admissions was selected for review, encompassing a total of 195 unique patients (5 patients had multiple high cost encounters). The mean cost (SD) for each admission was $49,923 CDN ($45,773). Approximately 73% of these costs were for direct patient care (mean (SD) $36,358 ($33,501)), of which the top components were: nursing care, special care units, and pharmacy costs (Table 2).

**Table 2:** Breakdown of average cost per categories for high cost admissions (*n* = 200)

Cost in CDN dollars Mean (SD)	
Total Cost	$49,923 (45,773)
Total Indirect Cost	$13,566 (12,625)
Total Direct Cost	$36,358 (33,501)
Direct Costs per costing Category	
Health Professionals	$2600 (4787)
Imaging	$1069 (1428)
Lab	$1923 (1709)
Nursing	$16,378 (16,735)
Operating room	$1192 (1819)
Surgical Implants	$907 (2821)
Post anaesthesia care unit	$307 (590)
Pharmacy	$3273 (4157)
Special Care Unit	$7502 (16,929)
Endoscopy	$72 (223)
Food Services	$1134 (1247)

Baseline characteristics are reported in Table 3. The mean age (SD) was 69 (15) years and 92/200 (46%) were male. The mean (SD) Elixhauser comorbidity score was 7.1 (6.6). The median (IQR) length of stay was 27 (18–48) days and 58/200 (29%) spent time in special care units with a median (IQR) ICU stay of 9 (6–14) days. One-third of admissions (33%; 65/200) included an ALC component, with a median (IQR) ALC time of 29 (17–40) days. In total, 62 admissions (31%) resulted in a discharge to long-term care. Almost half the discharges had more than one inpatient visit in the following year (88/200 (44%)), for an average of 15 additional days in hospital. About 14% (28/200) of admissions ended with in-hospital mortality.

**Table 3:** Baseline patient and encounter-level characteristics for high cost admissions (*n* = 200)

Variable		High cost admissions
		*n* = 200
Age at Admission – Mean (SD), years		69 (15)
Male		92 (46%)
Admitted route	Emergent	146 (73%)
	Urgent	20 (10%)
	Elective	34 (17%)
Elixhauser Comorbidity Score - Mean (SD)		7.1 (6.6)
Total LOS (days) - Median (IQR)		27 (18–48)
Acute LOS (days) - Median (IQR)		21 (14–31)
ICU Days	>0	58 (29%)
	Median (IQR)	9 (6–14)
ALC Days	>0	65 (33%)
	Median (IQR)	29 (17–40)
Total Cost (CDN) - Median (IQR)		$35,438 ($23,963–$54,075)
Discharge disposition	Died	28 (14%)
	Home	38 (19%)
	Home with supportive services	53 (27%)
	Other acute care facility	16 (8%)
	Long-term care	62 (31%)
	Other	3 (2%)
Reason for admission	Cancer	12 (6%)
	Complex medical	98 (49%)
	Elective surgery	28 (14%)
	Emergent surgery	28 (14%)
	Maternal	2 (1%)
	Social	9 (5%)
	Trauma	23 (12%)
Complications		119 (60%)
Services delay		78 (39%)
Disposition delay		105 (53%)
Inefficient clinical decision-making		25 (13%)
Inpatient encounters in 365 days post discharge	Total accumulated inpatient days - Mean (SD)	15 (33)
	0 visits	112 (56%)
	1 visit	51 (26%)
	2 visits	19 (10%)
	3 visits	12 (6%)
	4+ visits	6 (3%)

*Abbreviations*: *ALC* Alternate Level of Care, *ICU* Intensive Care Unit, *IQR* Inter-quartile Range, *LOS* Length of Stay, *SD* Standard Deviation

Of the factors identified in the qualitative research contributing to high-cost stays, complications were the most common contributor having occured in 60% of the charts reviewed (119/200). Half of the charts (53%; 105/200) reviewed were affected by disposition delays, while service delays occured in nearly 40% of the hospitalizations reviewed (78/200) and inefficient clinical decision-making was a contributor to cost in over 10% (25/200). Over half (55%) of cases had more than one of these contributors (110/200) while only 10% (19/200) had none (Table 3).

The reasons for admission were complex medical problems (98/200 (49%)), followed by emergent surgery/ies (28/200 (14%)), elective surgery/ies (28/207 (14%)), trauma (23/200 (12%)), cancer (12/200 (6%)), social (9/200 (5%)), and maternal (2/200 (1%)). The characteristics of patients with the four most common reasons for admission are reported in Table 4. Patients admitted because of complex medical problems had higher Elixhauser scores (mean (SD) 8.5 (6.6)) compared to patients admitted for other reasons. Patients admitted for elective surgery/ies had the shortest total length of stay (median (IQR) 16 (10–28) days) while patients admitted for trauma and emergent surgery had the longest length of stays relative to other admission types (*p* < 0.05). Patients admitted for elective surgery were also unique in that they had the fewest ALC days (0 days), fewer service delays (5/28 (18%)), and the fewest disposition delays (3/28 (11%)) compared to all other admission types (p < 0.05 for all comparisons). Admissions for trauma were the most likely to have disposition delays (20/23 (87%)), and had one of the highest median ALC days (median (IQR) of 27 (13–62) days).

**Table 4:** Subgroup analysis stratified by the four most common reasons for admission

Variable^a^		Reason for admission			
		Complex medical (*n* = 98)	Elective surgery (*n* = 28)	Emergent surgery (*n* = 28)	Trauma (*n* = 23)
Age at admission - Mean (SD), years		70 (15)	67 (12)	64 (14)	75 (15)
Male		44 (45%)	18 (64%)	15 (54%)	8 (35%)
Admitted route	Emergent	84 (86%)	5 (18%)	23 (82%)	23 (100%)
	Elective	5 (5%)	21 (75%)	2 (7%)	0 (0%)
	Urgent	9 (9%)	2 (7%)	3 (11%)	0 (0%)
Elixhauser Comorbidity Score - Mean (SD)		8.5 (6.6)	5.2 (5.7)	3.4 (4.1)	5.7 (5.5)
Total LOS (days) - Median (IQR)		24 (19–47)	16 (10–28)	31 (15–51)	29 (22–58)
Acute LOS (days) – Median (IQR)		21 (15–28)	16 (10–28)	25 (14–46)	21 (19–29)
ICU Days	>0	34 (35%)	6 (21%)	11 (39%)	4 (17%)
	Median (IQR)	9 (7–13)	7 (3–8)	8 (4–24)	5 (3–11)
ALC Days	>0	36 (37%)	0 (0%)	8 (29%)	12 (52%)
	Median (IQR)	28 (17–38)	0	18 (13–35)	27 (13–62)
Total Cost (CDN) – Median (IQR)		$34,680 (23,969–54,258)	$34,137 (23,844–45,713)	$41,738 (23,475–83,092)	$38,529 (23,958–53,893)
Discharge Disposition	Died	20 (20%)	1 (4%)	4 (14%)	2 (9%)
	Home	18 (18%)	9 (32%)	6 (21%)	0 (0%)
	Home with supportive services	22 (22%)	14 (50%)	7 (25%)	6 (26%)
	Other acute care facility	6 (6%)	2 (7%)	4 (14%)	3 (13%)
	Long-term care	29 (30%)	2 (7%)	7 (25%)	12 (52%)
	Other	3 (3%)	0 (0%)	0 (0%)	0 (0%)
Complications		62 (63%)	17 (61%)	19 (68%)	8 (35%)
Services delay		42 (43%)	5 (18%)	12 (43%)	14 (61%)
Disposition delay		54 (55%)	3 (11%)	15 (54%)	20 (87%)
Inefficient clinical decision-making		19 (19%)	2 (7%)	2 (7%)	2 (9%)
Inpatient encounters in 365 days post discharge	Total accumulated inpatient days Mean (SD)	14 (36)	12 (18)	16 (29)	18 (40)
	0 visits	59 (60%)	14 (50%)	14 (50%)	13 (57%)
	1 visit	22 (22%)	6 (21%)	7 (25%)	9 (39%)
	2 visits	9 (9%)	6 (21%)	2 (7%)	0 (0%)
	3 visits	5 (5%)	2 (7%)	4 (14%)	0 (0%)
	4+ visits	3 (3%)	0 (0%)	1 (4%)	1 (4%)

Note: Patients admitted for “cancer with chemotherapy”, “maternal”, or “social” reasons were not reported in this table due to low numbers (10 or fewer total encounters per category). Abbreviations: *ALC* Alternate Level of Care, *ICU* Intensive Care Unit, *IQR* Inter-quartile Range, *LOS* Length of Stay, *SD* Standard Deviation

^a^ All statistical testing across reasons for admission were statistically significant (based on either the chi-squared test for proportions, t-test for means, or Kruskal-Wallis test for medians). Therefore *p*-values for each estimate are not reported

## Discussion

Using an exploratory sequential mixed methods approach we found that while high cost admissions often involved older, medically complex patients, a large proportion of these admissions accumulated costs as a result of a medical complication, delays in disposition or medical services, or a delay due to inefficient decision-making. Further, half of the high cost admissions had at least two of these factors and occured more frequently in certain types of admissions. These findings suggest opportunities exist to improve outcomes and reduce costs for this diverse patient population.

Consistent with previous work we found that high-cost users are a heterogenous group [6, 8, 9, 24]. This heterogeneity is apparent when high cost users are stratified by their reason for admission. For example complex medical patients had high comorbidity burden and longer lengths of stay, whereas people admitted for elective surgery had lower comorbidity burden and shorter lengths of stay. This highlights the importance of targeting specific cost control interventions at specific populations; a blanket approach is unlikely to succeed.

The most common potentally modifiable contributor to cost in our study was medical complications. This finding is consistent with decades of research in patient safety [25–27] and, while not all complications are preventable our findings are a reminder of the ongoing work needed to improve patient safety. The aims of improving safety and reducing costs are not separate goals but one and the same. It should also be recognized that cost may be a surrogate for quality; patients with complications and delays in their care understandably have higher costs. Although we have addressed this research question from a cost perspective, all of the processes of care we have identified are quality metrics that are highly relevant from the patient perspective. Thus, addressing these quality measures will not only improve quality of care but will also have a big impact on patient expectation and experience.

Perhaps one of the most easily targeted contributors to cost is the 50% of patients with a disposition delay. Having appropriate discharge destinations in the community is important because often an unanticipated admission to hospital leads to a change in a person’s functional status resulting in the need for a more supported living environment [28–30]. Disposition delays are an issue for healthcare systems in many countries and has no single solution. One option, building more post-acute care beds, is expensive but may decrease disposition delays – which we have shown is one of the major contributors to high cost hospitalizations [31]. However, there is concern that because there are no clear guidelines about which patients benefit most from post-acute care these spaces would soon be filled with patients who may not be appropriate [32]. Another option that has shown promise to reduce disposition delays is better integration of health care services so that healthcare is delivered to patients regardless of their physical location [33].

Our study has a number of strengths including a mixed methods design that allowed for a detailed and rich characterization of the various factors that contribute to high cost encounters. There are few studies that have described high-cost populations in such depth, and this research allowed for the identification of key themes that can inform future research and interventions within this patient population [6–8]. However, this study should be interpreted in light of its limitations. First, we determined factors associated with increased costs based on chart review; therefore poor documentation by the care-team could result in under-reporting of certain factors. We minimized this possibility by looking at notes from all healthcare professionals including consultants and nursing staff. Second, many of our outcomes were defined dichotomously. For example, disposition delays were identified if discharge was delayed by greater than 24 h to address this issue. While this allowed us to determine the frequency of delays, it did not provide the magnitude of effect on overall cost. This decision was made because of the difficulty in attributing an exact number of additional days in hospital to a specific issue. Third, the lack of a comparator group of non-high cost admissions prevents us from determining the relative magnitude of each factor on the outcome of interest. Despite this limitation, our findings are hypothesis-generating and provide valuable insight for future large scale studies that aim to understand the contribution/importance of these common factors to high cost admissions. Finally, this study was limited to a single Canadian tertiary care setting and may not be generalizable to other jurisdictions. Nevertheless, we believe that our results are generalizable because our centre includes multiple hospitals with more than 1000 beds and our findings are consistent with the literature in this area.

## Conclusions

Medical complications, delays in discharge disposition, delays in medical services, and inefficient clinical decision-making are common and potentially modifiable factors that contribute to cost among high-cost hospital patients. While efforts to improve patient safety and integrate health services across care settings may require initial investment, since these are the major drivers of high cost hospitalization, they also have the potential to decrease cost in the long-term.

## Abbreviations

ALC: Alternate Level of Care
CIHI: Canadian Institute for Health Information
ICD-10-CA: International Statistical Classification of Diseases and Related Health Problems, 10th revision – Canada
ICU: Intensive Care Unit
IQR: Inter-quartile Range
SD: Standard Deviation
